# Synchronous isolated splenic metastasis from cancer of hepatic flexure of colon: A case report

**DOI:** 10.1097/MD.0000000000015016

**Published:** 2019-04-05

**Authors:** Huiying Zhao, Weixiang Zhong, Dong Chen, Xiaofei Cheng

**Affiliations:** a Department of Colorectal Surgery; b Department of Pathology, The First Affiliated Hospital, School of Medicine, Zhejiang University, Hangzhou, Zhejiang, China.

**Keywords:** colorectal cancer, isolated splenic metastases, multidisciplinary team, splenectomy

## Abstract

**Rationale:**

Isolated splenic metastasis from colorectal cancer is very rare, as metastatic colorectal cancer involving the spleen is usually a manifestation of widely disseminated disease. Splenectomy is the best therapeutic option for this entity and probably the only chance for radical cure.

**Patient Concerns:**

A 73-year-old male presented with abdominal distension and dark red bloody stool of 6-month duration.

**Diagnoses:**

Synchronous isolated splenic metastasis from colorectal cancer.

**Interventions:**

Based on multidisciplinary team (MDT) mode, the patient underwent the primary hepatic flexure tumor resection due to his poor general condition. One month after surgery the patient began treatment with Xelox (capecitabine 1000 mg/m^2^, oxaliplatin 130 mg/m^2^) every 3 weeks. The patient underwent isolated splenic metastasis resection successfully by laparoscopic after four courses of chemotherapy.

**Outcomes:**

The patient's postoperative course was uneventful and he completed four courses of postoperative chemotherapy using the original chemotherapy regimen Xelox (capecitabine 1000 mg/m^2^, oxaliplatin 130 mg/m^2^). The patient was subsequently followed up every 3 months and no signs of recurrence were noted in a recent examination.

**Lessons:**

To the best of our knowledge, this is the first case report of isolated splenic metastasis from colorectal cancer in China. It is also the first case in which treatment was overseen by an MDT. The possibility of splenic metastasis should be considered in cases in which colorectal cancer is associated with a splenic lesion, despite its rarity. Splenectomy and adjuvant chemotherapy are the optimal therapeutic approaches, as such an approach prolongs survival and palliates the disease.

## 1 Introduction

Isolated splenic metastasis from colorectal cancer is very rare, as metastatic colorectal cancer involving the spleen is usually a manifestation of widely disseminated disease.^[1]^ In a cadaver study, the incidence of splenic metastases from colorectal cancer was 2% of 1019 cases, but the incidence of isolated splenic metastasis was not reported.^[2]^ To the best of our knowledge, only 32 cases (5 synchronous and 27 metachronous cases) of isolated splenic metastasis from colorectal cancer have been reported in the English-language literature.^[3]^ Because synchronous splenic metastasis is very rare, preoperative diagnosis is difficult. Splenectomy is the best therapeutic option for isolated splenic metastasis and probably the only chance for radical cure. Some authors suggest that splenectomy may lead to long-term survival in patients with isolated splenic metastasis.^[4,5]^ Herein, we report the first case of isolated splenic metastasis from colorectal cancer in China. We diagnosed this rare clinical entity and resected the primary tumor and isolated splenic metastasis using a multidisciplinary team (MDT) approach. MDT approach is important for advanced colorectal cancer, especially for patients with synchronous metastasis.^[6,7]^

## 2 Case report

**Figure 1:**
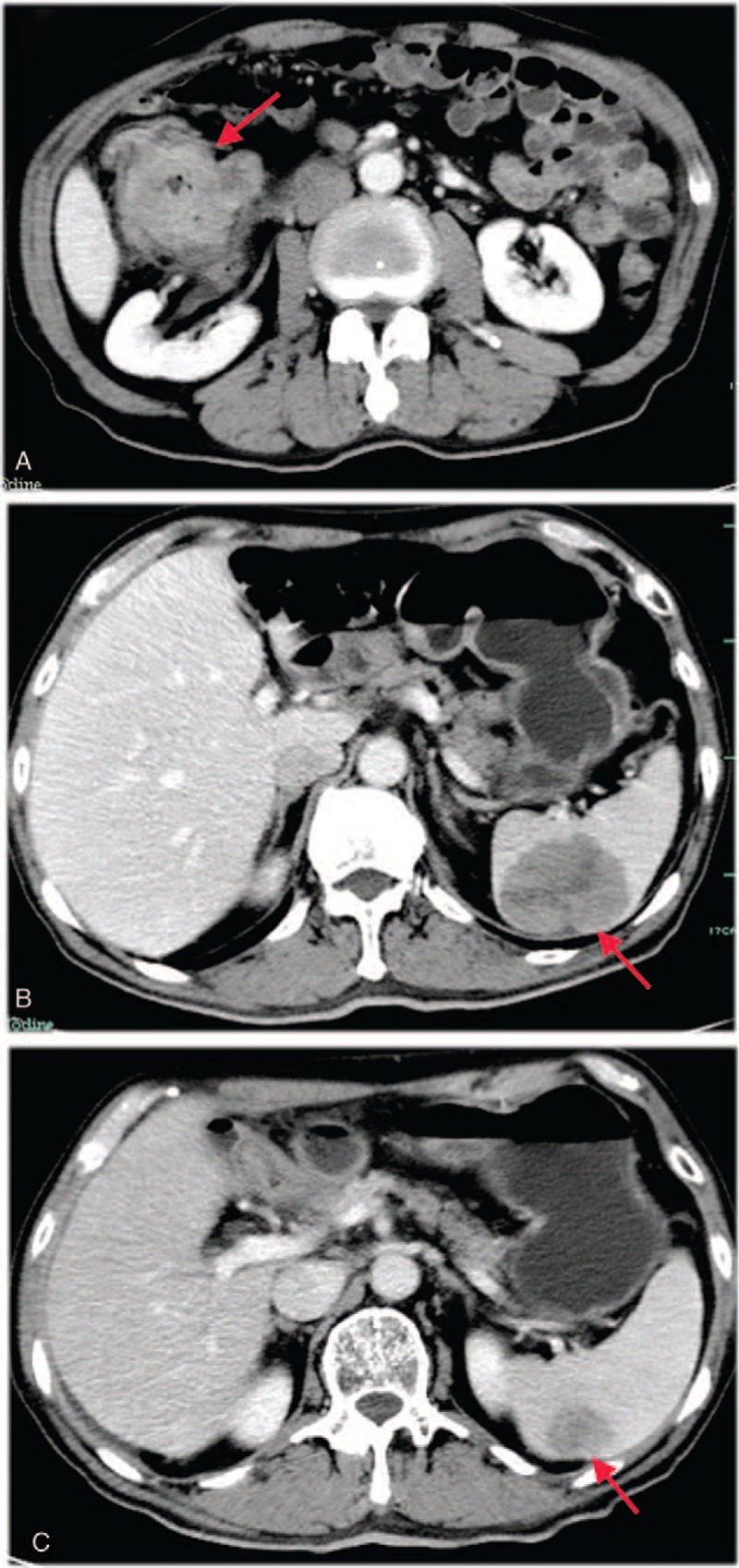
Whole abdominal computed tomography (CT). (A) a heterogeneous enhanced tumor in hepatic flexure; (B) a single low-density lesion of the spleen about 5.7 cm in diameter before primary treatment; (C) the spleen mass about 2.5 cm in diameter after 4 courses chemotherapy with Xelox (capecitabine, oxaliplatin).

**Figure 2:**
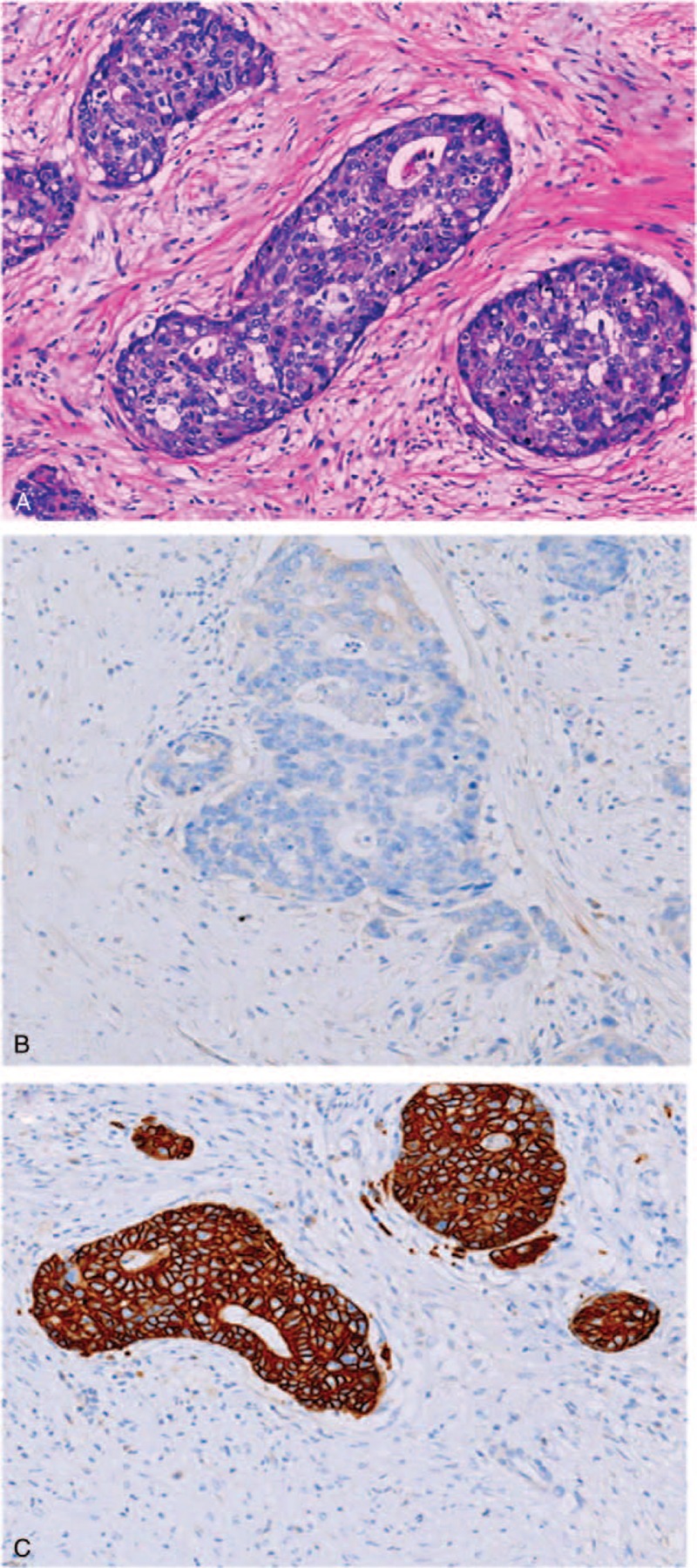
Histological findings of the tumor of the spleen. (A) Moderately-poorly differentiated adenocarcinoma (HE, ×100); (B) immunostaining for cytokeratin 7 showing a negative reaction (×100); (C) immunostaining for cytokeratin 20 showing a positive reaction (×100).

In October 2017, a 73-year-old male presented with abdominal distension and dark-red bloody stool of 6-month duration. He also complained of general fatigue and weight loss of 15 kg. He had no familial history of cancer, no prior pathological conditions, and no concomitant medication use. The patient's carcinoembryonic antigen (CEA) and hemoglobin levels were 6.9 ng/mL (0–5 ng/mL) and 101 g/L (131–175 g/L), respectively. The results of all other laboratory tests were normal, including cancer antigen 19 to 9 (CA 19–9), biochemical, and hematologic tests. Endoscopic examination revealed an obstructing neoplasm in the hepatic flexure, about 4.5 cm in diameter, with surface depression, erosion, and a propensity for bleeding. A biopsy of the lesion established a diagnosis of moderately differentiated adenocarcinoma. Whole-abdomen computed tomography (CT) revealed wall thickening of the hepatic flexure with proximal incomplete intestinal obstruction (Fig. 1A). The CT scan also revealed a single low-density lesion of about 5.7 cm diameter in the spleen (Fig. 1B). Genetic testing of the biopsy material indicated non-mutated *KRAS*, *NRAS*, and *BRAF* genes. After the first MDT discussion, due to the poor general condition of the patient, we decided to remove the primary lesion and biopsy the splenic mass during the operation. The biopsy indicated the presence of splenic metastasis from adenocarcinoma. The patient underwent a laparoscopic right hemicolectomy due to the histopathological finding of a moderately and poorly differentiated adenocarcinoma invading the serosa. Twenty-two lymph nodes were removed and 7 showed metastases (pT3N2M1, stage IV). One month later, the patient's CEA level had decreased to 3 ng/mL. The patient's postoperative recovery was uneventful, and 1 month after surgery he began treatment with Xelox (capecitabine 1000 mg/m^2^, oxaliplatin 130 mg/m^2^) every 3 weeks for 3 months. After 4 courses of Xelox therapy, the patient's clinical response was excellent, with II degree vomiting and no obvious bone marrow suppression or neurotoxicity. The diameter of the splenic lesion had decreased from 5.7 to 2.5 cm (Fig. 1C), and a partial response had been achieved after 4 courses of chemotherapy. In April 2018, based on a second MDT discussion, the patient underwent laparoscopic splenectomy. The histological findings showed that the splenic tumor was a moderately and poorly differentiated adenocarcinoma, similar to the tumor of the hepatic flexure (Fig. 2A). Negative staining for cytokeratin 7 and positive staining for cytokeratin 20 was consistent with splenic metastasis of an adenocarcinoma of the hepatic flexure (Fig. 2B and C). The patient's postoperative course was uneventful and he completed 4 courses of postoperative chemotherapy using the original chemotherapy regimen Xelox (capecitabine 1000 mg/m^2^, oxaliplatin 130 mg/m^2^). The patient's clinical response was good, with II degree vomiting, II degree bone marrow suppression, and II degree neurotoxicity. The patient was subsequently followed up every 3 months and no signs of recurrence were noted in a recent examination on January, 2019.

## 3 Discussion

Approximately 1 in 4 patients with colorectal cancer have metastases at the time of initial diagnosis.^[8]^ Metastases to the spleen from colorectal cancer in the absence of liver or lung involvement are extremely rare,^[9,10]^ possibly due to the anatomical and immunological characteristics of the tumor. The anatomical factors that restrict metastasis include the rhythmic contraction of the sinusoidal splenic architecture and the sharp angle of the splenic artery with the celiac axis.^[11]^ Moreover, immune surveillance in the spleen inhibits tumor cell proliferation.^[12]^ A PubMed search yielded only 32 cases of isolated solitary splenic metastasis from colorectal cancer (synchronous, 5 cases summarized in Table 1;^[13–16]^ metachronous, 27 cases) in the English language literature. We report here the sixth case in which an isolated splenic lesion was synchronous with colorectal cancer. A particularly interesting aspect of this case is its diagnosis and treatment by an MDT. The MDT is defined as “a group of people of different health-care disciplines, which meets together at a given time to discuss a given patient and who are each able to contribute independently to the diagnostic and treatment decisions about the patients.” The colorectal cancer MDT in our hospital includes colorectal surgeons, hepatobiliary surgeons, urological surgeons, thoracic surgeons, gynecologists, radiologists, radiation therapist, histopathologists, medical oncologists, and nurse specialist. An MDT approach may not only reduce the risk for perioperative morbidity and mortality but also improves long-term survival.^[17,18]^ Because of the poor general condition of the patient, the staging and resection of the primary and metastatic lesions were overseen by an MDT.

**Table 1:**
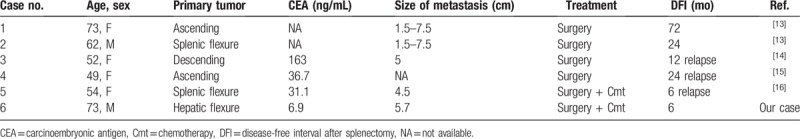
Summary of reported cases of synchronous isolated splenic metastasis from colorectal cancer.

Because the majority of patients with isolated splenic metastasis from colorectal cancer are asymptomatic, the condition is typically diagnosed through imaging tests and evaluation of CEA levels. CEA levels are elevated in more than 80% of such cases, to 4.6 to 223 ng/mL^3^; the level in our patient was 6.0 ng/mL. In addition, careful examination of an abdominal CT scan can facilitate early diagnosis of synchronous and metachronous splenic metastasis; therefore, this should be a priority.^[19]^ None of the cases reported to date were misdiagnosed. Overall, isolated splenic metastasis is relatively easy to diagnose.

Splenectomy is necessary in the presence of isolated metastases from colorectal cancer.^[16]^ Splenectomy was performed in all reported cases of isolated splenic metastasis from colorectal cancer, but only two cases were treated using a laparoscopic approach, as in our patient. Due to the risk for peritoneal dissemination, use of laparoscopy for splenic malignancies is controversial. However, laparoscopic splenectomy for splenic metastasis reportedly does not increase the rate of surgical complications, and survival ranges from 2 months to 11 years.^[5]^ Moreover, laparoscopic surgery for other abdominal tumors is not associated with a greater risk of surgical complications compared to conventional techniques.^[20,21]^ Thus, laparoscopic splenectomy for splenic metastasis is safe and reproducible, and yields outcomes superior to those of open surgery. The survival duration after splenectomy ranges from 3 to 84 months (mean, 22.5 months).^[3]^ Only 2 of 27 reported cases of metachronous splenic metastasis relapsed between 9 and 11 months, compared to 3 of 5 cases of synchronous splenic metastases.^[3,22]^ Chemotherapy is the appropriate treatment for isolated splenic metastases from colorectal cancer, particularly synchronous splenic metastases.^[23]^ Chemotherapy regimens for metastatic colorectal cancer include CapeOX/Xelox (capecitabine 1000 mg/m^2^, oxaliplatin 130 mg/m^2^), mFOLFOX6 (5-fluorouracil 2400 mg/m^2^, leucovorin 400 mg/m^2^, and oxaliplatin 85 mg/m^2^), and FOLFIRI (5-fluorouracil 2400 mg/m^2^, leucovorin 400 mg/m^2^, and irinotecan 180 mg/m^2^).^[18,24,25]^ Our patient underwent the primary hepatic flexure tumor and isolated splenic metastasis resection in stages due to his poor general condition. The interval between the 2 operations is about 3 to 4 months. Since this entity was advanced colorectal cancer (pT3N2M1, stage IV), our MDT team recommended that the patient underwent adjuvant chemotherapy first during the waiting period. Our patient completed perioperative chemotherapy using the regimen Xelox (capecitabine 1000 mg/m^2^, oxaliplatin 130 mg/m^2^).

In conclusion, we report the first case of isolated splenic metastasis from colorectal cancer in China; it is also the first case in which treatment was overseen by an MDT. Despite its rarity, the possibility of splenic metastasis should be considered in cases in which colorectal cancer is associated with a splenic lesion. Splenectomy and adjuvant chemotherapy is the optimal therapeutic approach; such an approach prolongs survival and palliates the disease. We report this rare clinical entity for the purpose of improving the management and survival of patients with isolated splenic metastases from colorectal cancer.

## Acknowledgments

We gratefully acknowledge the contributions made by the clinical nurse specialists, surgeons, radiologists, pathologists, and oncologists to the Colorectal MDT. In particular, we would like to thank: Jianjiang Lin, Wenbin Chen, and Yingsheng Wu.

## Author contributions

**Data curation:** Huiying Zhao, Weixiang Zhong.

**Investigation:** Dong Chen.

**Visualization:** Weixiang Zhong.

**Writing – Original Draft:** Huiying Zhao.

**Writing – Review & Editing:** Xiaofei Cheng.
